# Reducing corticosteroid phobia in pharmacy staff and parents of children with atopic dermatitis

**DOI:** 10.1007/s11096-021-01241-2

**Published:** 2021-02-13

**Authors:** Ellen S. Koster, Daphne Philbert, Xiang Zheng, Nila Moradi, Tjalling W. de Vries, Marcel L. Bouvy

**Affiliations:** 1 Division of Pharmacoepidemiology and Clinical Pharmacology, Utrecht Institute for Pharmaceutical Sciences, Faculty of Science, Utrecht University, PO Box 80082, 3508 TB Utrecht, The Netherlands; 2 Department of Pediatrics, Medical Centre Leeuwarden (MCL), Henri Dunantweg 2, 8934 AD Leeuwarden, The Netherlands

**Keywords:** Atopic dermatitis, Childhood, Corticophobia, Intervention, Parents, Pharmacy

## Abstract

*Background* Besides physicians, pharmacy staff has an important role to inform patients on appropriate medication use. However, they might also experience corticophobia themselves, affecting patient counseling and subsequently patient’s disease management. *Objective* Implementation of an intervention for pharmacy staff to improve knowledge and stimulate positive perceptions towards TCS use, in order to reduce corticophobia in pharmacy staff and parents of young AD patients. *Setting* Nine community pharmacies in the Netherlands. *Method* We developed an intervention consisting of education of pharmacy staff followed by counseling of parents. The intervention was implemented in pharmacies and intervention effectiveness was studied using a pre-post design with an intervention period of 3 months. At baseline and follow-up (3 months), pharmacy staff and parents completed a questionnaire. *Main outcome measure* Corticophobia, both beliefs and worries, measured with the TOPICOP questionnaire. Higher scores indicate a more negative attitude. *Result* Baseline and follow-up data were available for 19 pharmacy staff members and 48 parents who attended a counseling session in the pharmacy. In both groups there was as decrease in negative beliefs and worries towards TCS (*p* < 0.05). Mean total TOPICOP scores decreased from 42 to 35% and from 33 to 25% for parents and pharmacy staff respectively. *Conclusion* Our results show the prevalence of corticophobia among parents. Education of pharmacy staff and targeted patient counseling seems to be effective in reducing corticophobia.

## Impacts on Practice

- Pharmacy staff can help parents of children with AD to overcome corticophobia by providing targeted patient education and counseling.
- Pharmacy staff expressed concerns regarding use of corticosteroids. Their counseling should however not be influenced by their own prejudices, thus re-education might be necessary.
- With relatively limited time and effort, a clinically relevant improvement in pediatric AD treatment can be achieved.

## Introduction

Atopic dermatitis (AD) is a chronic inflammatory skin disorder that tends to flare periodically, with the highest prevalence in very young children [1, 2]. Symptoms vary widely between persons and include itch, redness of the skin and rash with sometimes open or crusted sores. Any area of the body may be affected, but symptoms usually affect areas where the skin flexes, e.g. inside the elbows, behind the knees and in front of the neck. There is no cure for AD, but symptoms can be treated effectively in most patients. Treatment consists of daily use of emollients (moisturizers) to keep the skin sufficiently hydrated and protected against irritants. Furthermore, it is important that patients try to avoid exposure to triggers that irritate the skin, such as taking long and warm baths or use of detergents. Although moisturizing is most of the times effective to keep the skin soft, temporary treatment with topical corticosteroids (TCS) is necessary during exacerbation to reduce inflammation, swelling and redness of the skin, so that the skin can heal [3]. TCS are applied directly onto the diseased skin, enhancing efficacy and reducing the risk of systemic side effects. TCS safety is high if correctly prescribed and administered [4, 5]. Despite the proven effectiveness of both moisturizers and TCS for treatment of AD, incorrect use and low adherence rates are common [6, 7]. An important reason for low TCS adherence are concerns about side effects and negative consequences of TCS use, so called ‘corticophobia’ [8, 9]. Strowd et al. [10] described the major hurdle of overcoming these adherence barriers, underlining the importance of clear patient education and counseling.

Patients and parents of young children are informed by prescribers, such as general practitioners, dermatologists and pediatricians about the effects and use of emollients and TCS. However, pharmacy staff, i.e. pharmacists and pharmacy technicians, are usually the last healthcare providers to see a patient before he or she goes home with (new) medicines. Improper counselling in the pharmacy, e.g. by stressing use of small amounts of cream can augment corticophobia and thereby interfere with the advice given by physicians [9]. Indeed, our previous research already showed that pharmacy staff do not always provide clear and correct instructions about application of creams in treatment of young AD patients. This may be the result of their own negative perceptions towards TCS use or lack of knowledge about proper eczema management [11]. In addition, other healthcare providers, including prescribers, involved in treatment of children with AD might also have negative views towards TCS use, which in turn also influences patient counseling and as a result patients’ medication use [12–14].

Based on previous findings [12–15] we speculated that improving pharmacy staff’s knowledge about treatment of AD would result in a more positive attitude towards TCS. This subsequently would improve counseling and lead to better treatment of patients with AD. Therefore, we developed an intervention aimed at pharmacy staff consisting of: (1) education of pharmacy staff followed by (2) targeted patient counseling.

### Aim of the study

We aimed to study the effect of this pharmacy intervention on corticophobia among both pharmacy staff and parents of young AD patients. In addition, we assessed the effect of the intervention on pharmacy staff’s knowledge about AD treatment and AD severity in pediatric patients.

### Ethics approval

The study was approved by the Institutional Review Board, department of Pharmaceutical Sciences, Utrecht University (reference number: UPOZ1705, data of approval: 1 November 2017. All procedures performed in studies involving human participants were in accordance with the ethical standards of the institutional research committee and with the 1964 Helsinki declaration and its later amendments or comparable ethical standards. All participants, pharmacy staff and parents, completed informed consent before start of the study.

## Method

### Setting and design

Community pharmacies affiliated with the Utrecht Pharmacy Practice network for Education and Research (UPPER) were approached to participate in the study [16]. We aimed to enrol at least 10 community pharmacies spread across the Netherlands, with at least one pharmacy staff member participating in the intervention. All participating pharmacies were visited by one of the two research assistants who gave additional instructions about intervention materials and further study procedures. Effectiveness of the intervention was studied using a pre-post design with an intervention period of 3 months, as this is usually the time between filling of two prescriptions.

### Participants

In these pharmacies all patients meeting the inclusion criteria: age ≤ 12 years, filling a prescription for any TCS (ATC group D07A) during the past 6 months with availability of a phone number in the pharmacy information system, were selected from the pharmacy information system. Parents of patients meeting these criteria received information about the study by postal mail. After two weeks a pharmacy staff member contacted them by phone and invited them to for a counselling session in the pharmacy. We expected to select 30–40 patients per pharmacy. Accounting for non-response and drop-out, we estimated to include 15–20 participants per pharmacy.

### Intervention

The intervention consisted of two parts: education of pharmacy staff followed by a counseling session for parents and children in the pharmacy. For the education of pharmacy staff we developed a website with information, such as background information about eczema and treatment, useful treatment guidelines and practical tips or tools to be used during patient counseling. Table 1 provides a summary of the information provided on the website. In addition, together with the Royal Dutch Pharmacist Society, we developed a knowledge assessment, consisting of 20 closed questions about eczema and treatment including feedback on (wrong) answers. Prior to the start of the patient counselling sessions, pharmacy staff was asked to study the materials on the website and take the online knowledge assessment. In each pharmacy, at least one pharmacy staff member involved in patient counseling was asked to take this test.

**Table 1:** Summary of website content

INTERVENTION PART 1 – preparation of patient counselling session	
**Improving knowledge of pharmacy staff** *Education of pharmacy staff about skin, skin care, atopic dermatitis, treatment and correct use and application of creams*	**Tool 1: Knowledge test atopic dermatitis** Pharmacy staff take the knowledge test before the first conversation with a parent **Tool 2: Overview courses and education** Overview of available courses and training for healthcare professionals about AD and treatment
**Stimulating collaboration between pharmacy staff and physicians** *Suggestions to improve collaboration:* *Align treatment advises (including patient advice on application, dosage *etc*.) with the general practitioner (GP)* *Provide the GP with relevant information about drug related problems notified in the pharmacy* *Provide patients who fill a prescription for a TCS also with a moisturizer. Notify the GP (afterwards) in case a prescription is needed* *Provide patients with samples of different moisturizers so they can find the best solution for them. Communicate this with the GP and ask for the prescription* *Organize a practical training session for pharmacy and GP staff in correct application of creams*	**Tool 3: Example of treatment application regimen** Align treatment advises with the GP and use the same advises and patient education materials **Tool 4: Referral to education materials to be used during pharmacotherapy consultations with the GP**

INTERVENTION PART 2 – patient counselling in the pharmacy	
**Identification of problem(s) in the pharmacy** *Check at the pharmacy counter if there are (practical) problems with drug use: attitudes about treatment and condition, side effects, patient needs* *Targeted selection of patients with potentially severe eczema or suboptimal treatment based on information from the pharmacy information system and invitation of patients for counseling session in the pharmacy*	**Tool 1: Instruction manual for patient selection from the pharmacy information system**
**Tailored counseling, based on problem or patient needs** *Suggestions for topics to address during patient counseling:* *Patient education about correct use (fingertip units) and warnings* *Advice about emollients (provide samples)* *Patient training in correct application of treatment (teach back)* *Providing patients with written or online materials* *Organize a patient information session about eczema and treatment in the pharmacy*	**Tool 2: Triage tool** Overview of questions to gain insight in causes of problems with treatment and follow-up actions **Tool 3: Leaflet with key elements of treatment** Pocket card including practical suggestions for treatment of AD (e.g. contact GP or pharmacist for questions) with space to write down treatment (application) advise **Tool 4: Patient education materials** Overview of materials to be used during patient counseling, such as websites for patients, booklets, flyers (written and online), patient organizations

Participating parents and children were invited for a counseling session in the pharmacy. During this encounter, they received information about eczema and its treatment, with special focus on treatment with moisturizers and correct use of TCS. The pharmacy staff member also addressed parental attitudes towards TCS use and practical (non-medical) advices for management of AD, such as trigger avoidance.

### Data collection

Pharmacy staff filled out an online questionnaire at baseline, before they took part in the educational part (studying the website and taking the knowledge test), and after three months. The baseline questionnaire contained sociodemographic questions (age, gender, work experience). Both the baseline and follow-up questionnaire included questions on corticophobia and knowledge about AD treatment. In addition, the follow-up questionnaire included questions about experiences with the intervention.

Parents were asked to fill out an online questionnaire before and three months after the counselling session in the pharmacy. The baseline questionnaire contained items about sociodemographic characteristics (age, gender of the child and ethnic background), AD severity and corticophobia. These measures were also included in the follow-up questionnaire. Furthermore, the follow-up questionnaire included questions to evaluate the counselling session. Data were collected between March and August 2018.

### Primary outcome: corticophobia

We used the TOPICOP questionnaire to assess TCS corticophobia [17]. This questionnaire has been used in the Dutch healthcare setting before [13]. This questionnaire includes 12 items and assesses both worries and beliefs towards TCS use. All questions are scored on a 4‐point scale varying from “totally disagree” to “totally agree” (score 0‐3). The six items concerning worries add up to a sum score between 0 and 18 for worries; the other six items, concerning beliefs, add up to a sum score between 0 and 18 for beliefs. These two scores together add up to the total TOPICOP score, ranging from 0 to 36, expressed as a percentage. Higher scores correspond to more severe corticophobia.

### Secondary outcome: knowledge

Knowledge of pharmacy staff was assessed with 10 closed questions from the knowledge test described above, including questions about eczema symptoms in children, lifestyle advice and preventive measures, TCS dosage, fingertip units, application of TCS and emollients, the mechanism of action and adverse effects of TCS. All correct answers received one point, resulting in a score ranging between 0 and 10.

### Secondary outcome: AD severity

The Patient Oriented Eczema Measure (POEM), a 7-item questionnaire was used to assess patient-reported severity of AD [18]. The POEM includes items about the frequency of symptoms such as dry skin, itching, and sleep disturbance because of eczema during the past week. All items are scored on a 5-point scale varying from “no days” to “every day” (score 0–4). The items add up to a sum score ranging from 0 to 28, with higher scores indicating a greater frequency of AD symptoms and sleep disturbance. A score ≥ 17 indicates (very) severe eczema symptoms, a score of 3–16 mild to moderate symptoms and a score between 0 and 2 indicates clear or almost clear eczema.

### Data analysis

Descriptive statistics, including check for normality, were calculated for all variables. Differences between baseline and follow-up were analyzed using paired sample t-test. P-values < 0.05 were considered statistically significant. All analyses were performed using SPSS version 25.0 (IBM corp. Armonk, NY).

## Results

### Study population

A total of 32 pharmacy staff members from 9 community pharmacies agreed to participate and completed the baseline questionnaire. Counseling sessions were performed by 19 of them. A total of 473 patients were invited from the 9 pharmacies for participation in the study. The most important reason for not willing to participate was not having AD, but another disease (e.g. impetigo or a once-only rash) for which use of TCS was indicated. In total, 91 parents filled out the baseline questionnaire of which 69 attended the counseling session in the pharmacy. Of these, 48 parents completed the follow-up questionnaire. The characteristics of the participants are shown in Table 2.

**Table 2:** Characteristics of pharmacy staff and parents who completed both questionnaires

*Pharmacy staff (N* = *19)*	
Female gender, % (n)	100 (19)
Age, mean (SD)	40.7 (13.4)
Function	
Pharmacy technician	68.4 (13)
Pharmacist	31.6 (6)
*Patients and parents (N* = *48)*	
Female gender child, % (n)	45.8 (22)
Age child, mean (SD)	3.6 (3.5)
Native Dutch background, % (n)	85.5 (42)
Medication use	
Emmolient	89.6 (43)
TCS	93.8 (45)
Responsible for application of creams	
Mother	64.6 (31)
Father	2.1 (1)
Both parents	29.2 (14)
Child	4.2 (2)

### Corticophobia

**Fig. 1:**
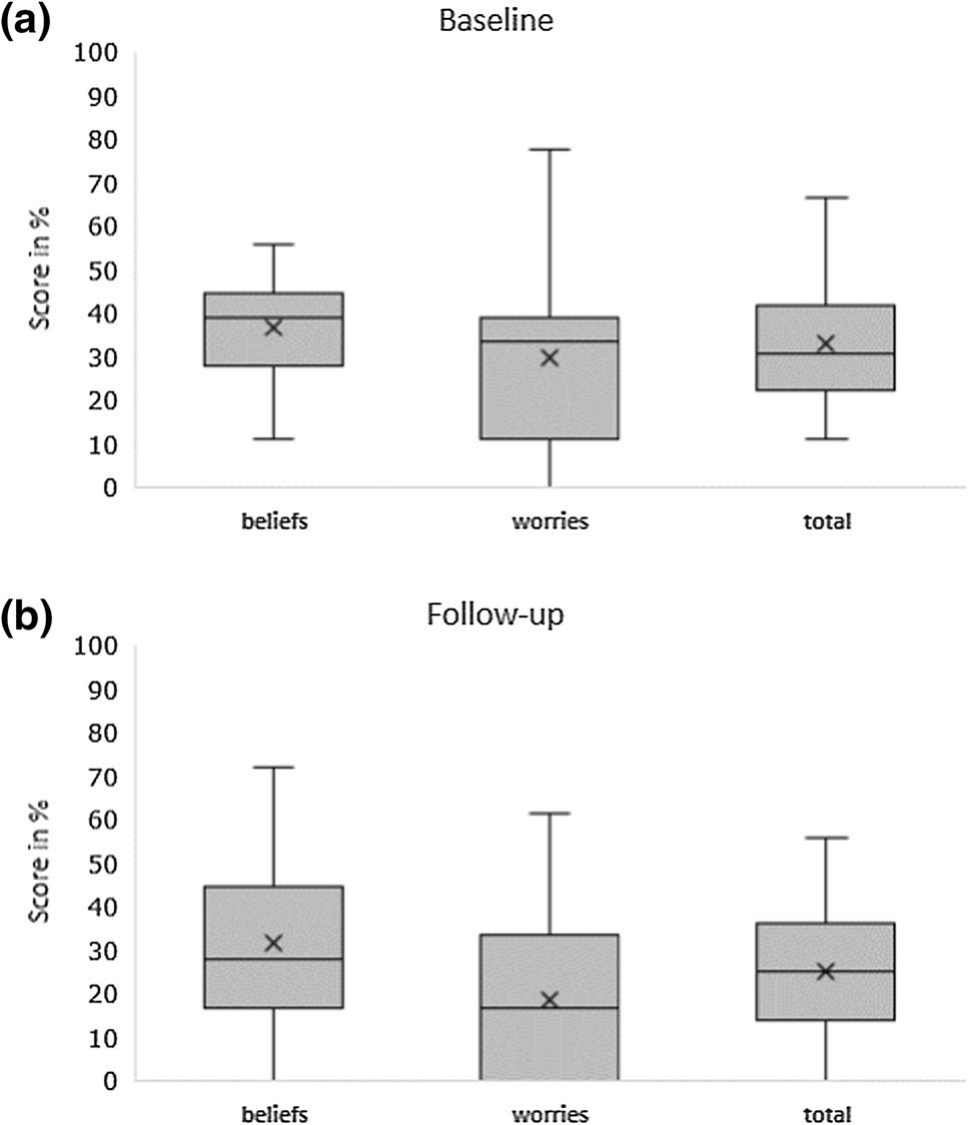
Box plots showing sum scores of the TOPICOP questionnaire for pharmacy staff at baseline (**a**) and follow-up (**b**)

**Fig. 2:**
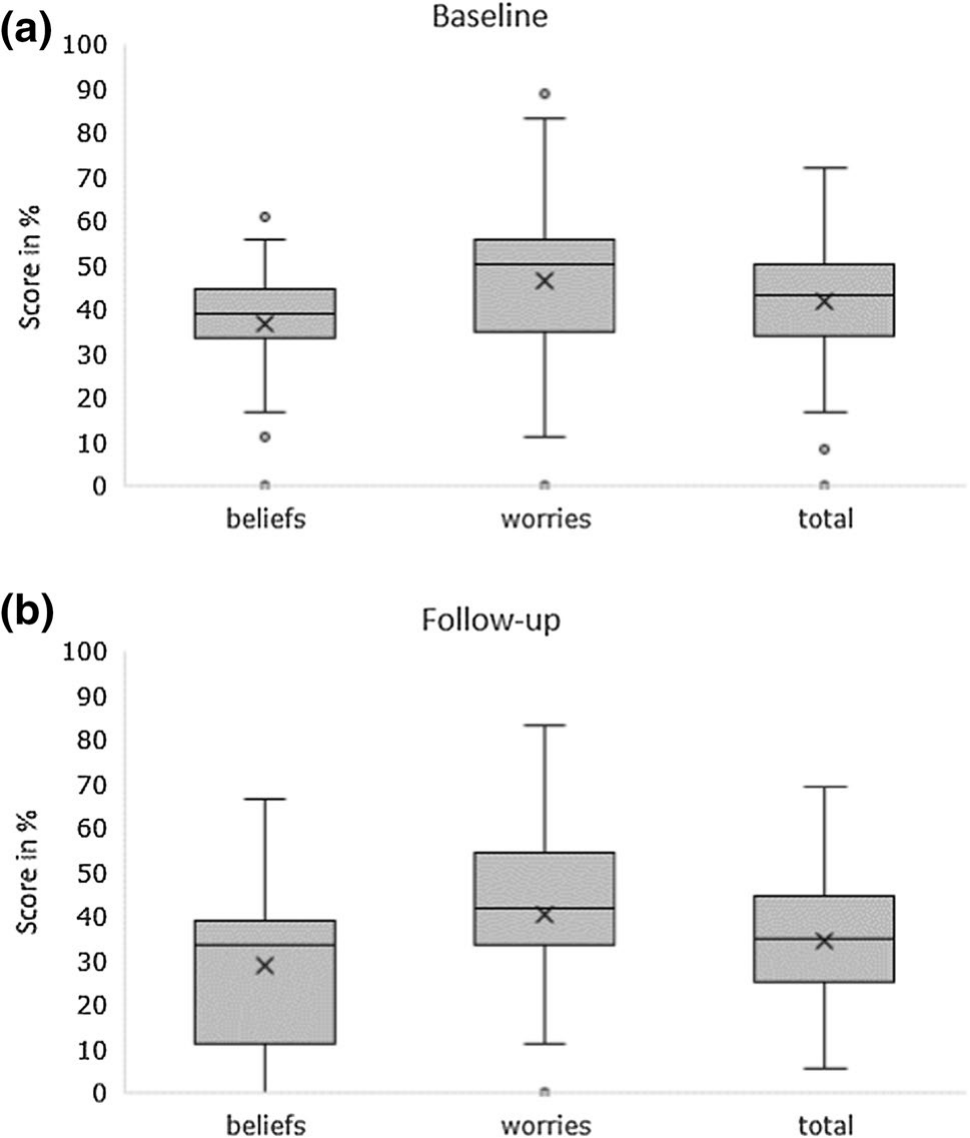
Box plots showing sum scores of the TOPICOP questionnaire for parents at baseline (**a**) and follow-up (**b**)

Figure 1 shows the box plots with the sum scores of the TOPICOP questionnaire for pharmacy staff before and after the intervention. The mean total TOPICOP scores were significantly higher at baseline compared to scores at follow-up (33.2% vs. 25.1%, *p* = 0.012). The decrease in total scores was mainly due to a decrease in worries at follow-up (29.5% vs. 18.7%, *p* = 0.014). Figure 2 shows the TOPICOP scores for parents before and after the intervention. The mean total scores were higher at baseline compared to scores at follow-up (41.6% vs. 34.5%, *p* = 0.005).

### Secondary outcomes

**Fig. 3:**
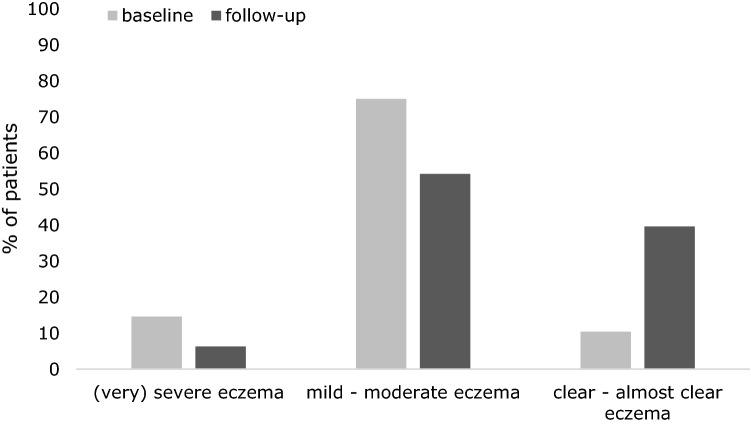
Parental reported atopic dermatitis severity at baseline and follow-up (POEM-scores): score ≥ 17 indicates (very) severe eczema symptoms, score 3–16 indicates mild to moderate symptoms and scores between 0 and 2 indicates clear or almost clear eczema

Knowledge about eczema and treatment among pharmacy staff increased from baseline to follow-up 7.3 ± 1.7 to 8.4 ± 1.5 (*p* = 0.052). Figure 3 shows the POEM scores at baseline and follow-up. At follow-up the percentage of patients with clear or almost clear eczema is significantly higher compared to baseline (39.6% vs. 10.4%, *p* < 0.001).

### Experiences with intervention

All pharmacy staff mentioned the intervention, both the website and the counseling session in the pharmacy, to be useful. They gained more insight in problems that patients and parents experienced. Also, parents were in general positive about the counseling session in the pharmacy. The majority (81.3%) mentioned the conversation to be useful and 45.8% mentioned they started using the treatment differently afterwards (e.g. more frequent use of emollients and increased application of TCS, based on FTU).

## Discussion

Corticophobia is present both among parents of young AD patients and pharmacy staff. Education of pharmacy staff followed by targeted patient counseling, effectively reduced corticophobia both among staff and parents. Furthermore, pharmacy staff’s knowledge about AD and treatment increased and patients seemed to experience less severe symptoms.

In line with previous studies, the parents in our study had negative perceptions towards TCS use [13, 15]. The total TOPICOP score in our study was 42%, similar to the score of 44% in the study of Bos et al. [13]. Parents mainly expressed their worries about side effects and negative consequences of (long-term) TCS use. Capozza et al. described worries about side effects as one of the top reasons for parents to deviate from treatment advices given by the physician [19]. These kind of worries can influence adherence and proper use of TCS. Incorrect use of treatment may exacerbate AD symptoms or side effects, resulting in decreased quality of life. Motivating parents to use treatment is thus of importance. Informing parents or caregivers about correct use of treatment and providing them with up-to-date information about effectiveness and safety, may be effective in improving perceptions towards medicines and thereby increasing adherence rates [20].

Parents receive information about AD and its treatment mainly from prescribing dermatologists, general practitioners and pediatricians [8]. They have an important role in providing parents with correct information and supporting them with medication use. Previously, Bos et al. [13] showed corticophobia among healthcare providers involved in treatment of pediatric patients with AD. Their study showed scores of 31% for pediatricians and public health physicians and a score of 39% for general practitioners. In line with these previous findings, we showed corticophobia among pharmacists and pharmacy technicians. This corticophobia can affect the perspective of parents towards medication use [21].

The total TOPICOP score for pharmacy staff in our study was 33%. Lambrechts et al. [14] described similar levels of corticophobia among pharmacists and suggested to re-educate healthcare providers, as counseling should not be influenced by their own prejudices. Interventions targeted at the sources of TCS phobia and focusing on patient education and counseling seem to be useful [8]. Lee and coworkers [22] showed for example that a simple educational intervention, a 10–15 min patient counseling session about TCS by dermatologists, improved patient compliance. In addition, other training pharmacy programs have shown to be effective in improving pharmacy staffs’ knowledge and skills with positive impact on patient outcomes [23–26]. Kang et al. [27] showed that an increase in community pharmacists’ knowledge level about topical corticosteroids positively associated the quality of practice.

To our knowledge this is the first pharmacy intervention study focusing on improvement of treatment in patients with AD. Strong points of our study are the relatively simple intervention strategy, which facilitates implementation in daily practice. We showed that with relatively limited time and effort, a clinically relevant improvement in pediatric AD care can be achieved. In addition, both pharmacy staff and parents were positive about the intervention. The developed tools were useful for pharmacy staff and parents value the attention and advice given by the pharmacy staff.

Our follow-up time was relatively short, so we only assessed short-term effects of our educational intervention. For a sustainable effect, regular training or attention of pharmacy staff is necessary to maintain awareness around optimal treatment of atopic dermatitis. In addition, it is important to address this regular during patient consultations.

We used online questionnaires for data collection. This is an efficient way of collecting data, as data are entered directly by participants in an online database and participants can fill out the questionnaire whenever and wherever they want to. A limitation might be the use of self-reported measurements, resulting in a potential desirability bias. However, pharmacy staff did not get insight in answers of parents and research staff did not attend the counselling session. Furthermore, attitudes towards medication (corticophobia) can only be measured using self-report. We used the TOPICOP questionnaire which has shown successfully before in different settings [13–15, 28]. However, there is no officially validated Dutch version of the questionnaire. However, we only used it for comparison in groups and used the questionnaire provide by Bos et al. The mean score we found in our study was similar to the scores presented by Bos et al. [15].

Another limitation is the relatively low response rate and high drop-out rate, 69 patients were part of the intervention, but for only 48 of them we received follow-up data. We aimed to enrol about 20 patients per pharmacy, which we unfortunately did not reach. This is partly due to our inclusion criteria, “dispensing of a TCS in the past 6 months”. As the pharmacy information system does not include information about the indication for use, TCS could also be prescribed for other treatment purposes and not only AD. This is one of the reasons for the loss of a part of our selected patients. Furthermore, only half of the parents who filled out the baseline questionnaire and attended the counseling session responded to the follow-up questionnaire. This may be due to limited time of young (working) parents. Besides that, loss to follow-up is common in longitudinal study designs in which repeated actions of the participants are expected. It is also possible that parents who had concerns towards TCS were more interested in participation in the study. Thus some degree of selection bias might have occurred. Furthermore, data were collected only during spring and summer, thus we could not take possible seasonality of symptoms into account. Some studies report peaks in AD symptoms during this period [29], whilst Kramer et al. [30] reported that symptoms and seasonal influences depend on the individual. In their study some children reported more severe symptoms in winter (temperature based) whilst other experiences more severe symptoms spring (allergy induced). Environmental factors, such as swimming and high temperature during summer might also influence occurrence of symptoms during this season.

## Conclusion

We can conclude corticophobia among parents of patient with AD exists, however we cannot be explicit about the magnitude of the problem. A relatively simple intervention seems to be effective in modifying both parental and pharmacy staff’s perceptions. Dermatologists, pediatricians and other physicians caring for children with AD should seek cooperation with pharmacists. Pharmacy staff can help parents of children with AD to overcome corticophobia by providing targeted patient education and counseling. These efforts can improve treatment of AD and thereby patients quality of life.

## References

[1] Dirven-Meijer PC, Kock CA, Nonneman MMG (2014). NHG-standaard eczeem. Huisarts Wet.

[2] Flohr C (2011). Recent perspectives on the global epidemiology of childhood eczema. Allergol Ummunopathol Madr.

[3] van Zuuren EJ, Fedorowicz Z, Christensen R, Lavrijsen A, Arents BWM (2017). Emollients and moisturisers for eczema. Cochrane Database Syst Rev..

[4] Youngh-Mi A, Hwang A, Jun K (2019). Real-world safety evaluation of topical corticosteroid use: a community pharmacy-based, prospective, observational study. Basic Clin Pharmacol Toxicol.

[5] Chiricozzi A, Comberiati P, D’Auria E, Zucottu G, Peroni DG (2020). Topical corticosteroids for pediatric atopic dermatitis: thoughtful tips for practice. Pharmacol Res.

[6] Brown KL, Krejci-Manwaring J, Tusa MG (2008). Poor compliance with topical corticosteroids for atopic dermatitis despite severe disease. Dermatol Online J.

[7] Krejci-Manwaring J, Tusa MG, Carroll C (2007). Stealth monitoring of adherence to topical medication: adherence is very poor in children with atopic dermatitis. J Am Acad Dermatol.

[8] Li AW, Yin ES, Antaya R (2017). Topical corticosteroid phobia in atopic dermatitis: a systematic review. JAMA Dermatol.

[9] Mueller SM, Itin P, Vogt DR (2017). Assessment of “corticophobia” as an indicator of non-adherence to topical corticosteroids: a pilot study. J Dermatolog Treat.

[10] Strowd LC, Feldman SR (2020). Overcoming poor adherence is a major hurdle to managing atopic dermatitis. Br J Dermatol.

[11] Linn AJ, van Weert JC, Schouten BC, Smit EG, van Bodegraven AA, van Dijk L (2012). Words that make pills easier to swallow: a communication typology to address practical and perceptual barriers to medication intake behavior. Patient Prefer Adherence.

[12] Koster ES, Philbert D, Wagelaar KR, Galle S, Bouvy ML (2019). Optimizing pharmaceutical care for pediatric patients with dermatitis: perspectives of parents and pharmacy staff. Int J Clin Pharm.

[13] Bos B, Antonescu I, Osinga H, Veenje S, de Jong K, de Vries TW (2019). Corticosteroid phobia (corticophobia) in parents of young children with atopic dermatitis and their health care providers. Pediatr Dermatol.

[14] Lambrechts L, Gilissen L, Morren MA (2019). Topical corticosteroid phobia among healthcare professionals using the TOPICOP Score. Acta Derm Venereol.

[15] Veenje S, Osinga H, Antonescu I, Bos B, de Vries TW (2019). Focus group parental opinions regarding treatment with topical corticosteroids on children with atopic dermatitis. Allergol Immunopathol (Madr).

[16] Koster ES, Blom L, Philbert D, Rump W, Bouvy ML (2014). The Utrecht Pharmacy Practice network for Education and Research: a network of community and hospital pharmacies in the Netherlands. Int J Clin Pharm.

[17] Moret L, Anthoine E, Aubert-Wastiaux H (2013). TOPICOP©: a new scale evaluating topical corticosteroid phobia among atopic dermatitis outpatients and their parents. PLoS ONE.

[18] Charman CR, Venn AJ, Williams HC (2004). The patient-oriented eczema measure: development and initial validation of a new tool for measuring atopic eczema severity from the patients' perspective. Arch Dermatol.

[19] Capozza K, Schwartz A (2020). Does it work and is it safe? Parents' perspectives on adherence to medication for atopic dermatitis. Pediatr Dermatol.

[20] Johnson MC, Pona A, Adler-Neal AL, Kesty C, Cline A, Feldman SR (2020). Assessing the effect of clinical trial evidence and anecdote on caregivers' willingness to use corticosteroids: a randomized controlled trial [formula: see text]. J Cutan Med Surg Jan/Feb.

[21] Horne R, Chapman SC, Parham R, Freemantle N, Forbes A, Cooper V (2013). Understanding patients’ adherence-related beliefs about medicines prescribed for long-term conditions: a meta-analytic review of the necessity-concerns framework. PLoS ONE.

[22] Lee JY, Her Y, Kim CW, Kim SS (2015). Topical corticosteroid phobia among parents of children with atopic eczema in Korea. Ann Dermatol.

[23] Ceulemans M, Liekens S, van Calsteren K, Allegaert K, Foulon V (2020). Impact of a blended learning program on community pharmacists’ barriers, knowledge, and counseling practice with regard to preconception, pregnancy and lactation. Res Social Adm Pharm.

[24] Showande SJ, Edidion NO (2020). Impact of pharmacists’ training on oral anticoagulant counseling: a randomized controlled trial. Patient Educ Couns.

[25] Battaglia JN, Kieser MA, Bruskiewitz RH, Pitterle ME, Thorpe JM (2012). An online virtual-patient program to teach pharmacists and pharmacy students how to provide diabetes-specific medication therapy management. Am J Pharm Educ.

[26] Liekens S, Vandael E, Roter D, Larson S, Smits S, Laekeman G, Foulon V (2014). Impact of training on pharmacists’ counseling of patients starting antidepressant therapy. Patient Educ Couns.

[27] Kang MJ, Park JH, Park S (2020). Community pharmacists' knowledge, perceptions, and practices about topical corticosteroid counseling: a real-world cross-sectional survey and focus group discussions in Korea. PLoS ONE.

[28] Stalder JF, Aubert H, Anthoine E (2017). Topical corticosteroid phobia in atopic dermatitis: international feasibility study of the TOPICOP score. Allergy.

[29] Fleischer AB (2019). Atopic dermatitis: the relationship to temperature and seasonality in the United States. Int J Dermatol.

[30] Krämer U, Weidinger S, Darsow U, Möhrenschlager M, Ring J, Behrendt H (2005). Seasonality in symptom severity influenced by temperature or grass pollen: results of a panel study in children with eczema. J Investig Dermatol.

